# Supplementary material for: Variations in the Distribution of Chl-a and Simulation Using a Multiple Regression Model
Source: Int J Environ Res Public Health. 2019 Nov 18;16(22):4553. doi: 10.3390/ijerph16224553 (PMC6888353; doi:10.3390/ijerph16224553)
Supplement: Supplementary file 1 [file ijerph-16-04553-s001.pdf]

Table S1. Supporting Information.

Collinearity statistics of the five water quality variables derived from a multiple linear regression using a stepwise method.

				Variance Proportions					
Model	D	Eigenvalue	CI	Constant	DOM	DO	TP	WT	pH
1	1	1.948	1.000	.03	.03				
	2	.052	6.108	.97	.97				
2	1	2.932	1.000	.00	.01	.00			
	2	.067	6.613	.01	.95	.01			
	3	.001	48.579	.99	.04	.99			
3	1	3.739	1.000	.00	.00	.00	.01		
	2	.228	4.048	.00	.00	.00	.34		
	3	.031	10.914	.01	.86	.00	.55		
	4	.001	57.891	.99	.14	1.00	.10		
4	1	4.585	1.000	.00	.00	.00	.00	.01	
	2	.241	4.366	.00	.00	.00	.32	.07	
	3	.142	5.685	.00	.01	.00	.01	.79	
	4	.031	12.101	.01	.84	.00	.52	.00	
	5	.001	69.079	.99	.14	1.00	.15	.14	
5	1	5.446	1.000	.00	.00	.00	.00	.00	.00
	2	.321	4.117	.00	.00	.00	.17	.01	.02
	3	.187	5.395	.00	.01	.00	.08	.30	.01
	4	.032	13.098	.00	.87	.00	.55	.00	.01
	5	.013	20.307	.05	.01	.01	.16	.39	.78
	6	.001	82.817	.95	.10	.99	.04	.30	.18

D denotes dimension; CI means the condition index
